# Memory Distortion for Traumatic Events: The Role of Mental Imagery

**DOI:** 10.3389/fpsyt.2015.00027

**Published:** 2015-02-23

**Authors:** Deryn Strange, Melanie K. T. Takarangi

**Affiliations:** ^1^John Jay College of Criminal Justice, The City University of New York, New York, NY, USA; ^2^Flinders University, Adelaide, SA, Australia

**Keywords:** imagery, intrusions, source monitoring, traumatic memory

Trauma memories – like all memories – are malleable and prone to distortion. Indeed, there is growing evidence – from both field and lab-based studies – to suggest that the memory distortion follows a particular pattern. People tend to remember more trauma than they experienced, and those who do, tend to exhibit more of the “re-experiencing” symptoms associated with post-traumatic stress disorder (PTSD). Our own research suggests that the likely mechanism underlying that distortion is a failure in people’s source monitoring. After a traumatic experience, intentional remembering (effortful retrieval) and unintentional remembering (intrusive mental imagery) can introduce new details that, over time, assimilate into a person’s memory for the event. We believe that understanding the role these factors play in distorting people’s memories for traumatic experiences is both theoretically and practically important, particularly given their potential role in influencing people’s recovery.

## Memory Distortion for Traumatic Events: The Role of Mental Imagery

People’s memories for traumatic events are – like their memories for more mundane events – easily distorted. Importantly, memory distortion for traumatic events appears to follow a particular pattern: people tend to remember more trauma than they experienced, a phenomenon referred to as “memory amplification.” Unfortunately, memory amplification carries real consequences: the more amplification people demonstrate, the more likely they are to report the “re-experiencing” symptoms associated with PTSD, such as intrusive thoughts and images [e.g., (1, 2)]. Our research program focuses on the mechanism by which memory amplification occurs. Specifically, we suspect people confuse the information generated *after* a traumatic event – both intentionally, for example, via conversation with others, and unintentionally, for example, via intrusive imagery – with what really occurred during the event. Put another way, we suspect the mechanism underlying memory amplification is a failure in people’s *source monitoring* that ultimately results in memory distortion (3, 4). In this review, we provide an overview of the source monitoring framework [SMF; (3, 4)], the evidence for traumatic memory distortion, and the role that we propose source monitoring errors, particularly imagery-based errors, play in promoting traumatic memory distortion.

First, let us briefly outline the tenets of the SMF (3, 4). Put simply, the SMF states that memory distortion occurs because we do not store our memories with a label specifying the origins of each individual detail. Generally this approach is an appropriate use of our capacity-limited cognitive resources and we employ simple heuristics to judge the origins of a particular detail or entire memory. However, sometimes those heuristics fail us. For example, event details that have been repeatedly or vividly imagined can come to mind more easily over time, and – if there is no trace of the effort that went into imagining those details – people can easily mistake the accompanying sense of familiarity for the familiarity that we know accompanies genuine recollection (5). A significant body of research has investigated the factors that make source monitoring more or less difficult, and thus source monitoring errors more or less likely to occur (3, 4). Many of those factors are an issue for traumatic experiences.

For example, traumatic events are highly likely to be rehearsed extensively in an intentional manner: victims will often make a statement to police, be exposed to media footage, and engage in conversations with other friends, family, doctors, or therapists (6). Each rehearsal opportunity comes with the potential for the inadvertent suggestion of misleading details [e.g., (3, 4, 7, 8)]. In addition, traumatic experiences are also frequently rehearsed in unintentional ways via intrusive images, thoughts, and memories; the “re-experiencing symptoms” typically associated with PTSD [e.g., (9)]. Sometimes, those thoughts and images will reflect genuinely experienced aspects of the event; sometimes, however, they may be memory traces of similar events witnessed in the news or entertainment media. In either case, people may inadvertently generate additional imagery relating to those traces that fits with the experienced event. Critically, over time, those non-experienced thoughts and images may become just as familiar as those that were experienced, increasing the likelihood of source monitoring errors (3, 4).

Several lines of converging evidence now document that people are susceptible to memory distortion for experiences of trauma, regardless of whether that trauma is a single event (such as a motor vehicle accident) or a sustained stressful experience that might involve multiple trauma types (such as military deployment). One line of research examines the impact of an external source of suggestion, such as suggestive questioning, on peoples’ memories for surprising, traumatic, and public events [e.g., (7, 8, 10–17)]. For example, Nourkova et al. (11) convinced people that they had witnessed a non-existent wounded animal in the film footage of the Moscow apartment bombings [(10–14); September, 1999]. Similarly, Crombag et al. (8) led participants to believe they had seen the moment an El Al Boeing 747 crashed into an apartment building, killing 43 people. Although there was no film of the crash, there was considerable media coverage of the aftermath. Indeed, participants often elaborated on the original suggestion (e.g., the plane was already burning when it crashed). Importantly, and in line with the SMF, Crombag et al. opined that traumatic events might be *more* susceptible to memory distortion than benign events because they typically provide more avenues for mental imagery, which can make source monitoring more difficult, and thus source monitoring errors more likely to occur (3, 4).

A second line of research with real victims of personal trauma examines how they remember their traumatic experience over time. These studies demonstrate that such victims often come to report being exposed to traumatic events they did not initially report experiencing (9, 18–25). For example, Southwick et al. (1) asked Desert Storm veterans at 1 month and 2 years after their return from service, whether certain events occurred during that service (e.g., sniper fire). They found 88% of veterans changed their response to at least one event; 61% changed more than one. Importantly, the majority of those changes were from “no, that did not happen to me” to “yes, that happened to me,” what has been termed “memory amplification.” How can we explain the change? One possibility is that the veterans were also exposed to external sources of suggestion during the intervening period. Additionally, the intrusive re-experiencing symptoms that typically accompany trauma exposure may have stimulated the production of other thoughts and images related to war-time experiences. In support of this possibility, Southwick et al. reported that the more severe the veteran’s re-experiencing symptoms, the more likely they were to exhibit memory amplification [see also Ref. (19, 21, 23)].

Our research provides a third line of evidence for the existence of traumatic memory distortion and the role mental imagery plays in that distortion. In our research, we have systematically examined the influence of source monitoring errors using a laboratory-based trauma analog. In one study, we showed participants a series of film clips depicting a fatal car accident (2). Each clip was separated by 2 s of blank screen, which allowed us to remove some scenes from the film. The next day participants returned to the laboratory for a surprise recognition memory test – comprised of scenes they had seen the day before (“old”), scenes we had removed from the original film (“missing”), and scenes depicting other road settings (“new”). The participants’ job was to identify whether each clip was old or new and how confident they were in that decision. Importantly, we divided the missing clips into *cruxes* (scenes critical to the film’s meaning; e.g., a child screaming for her parents) – which were also rated as the most traumatic scenes – and *non-cruxes* (more peripheral scenes; e.g., the arrival of a rescue helicopter). We found that participants were very good at recognizing what they had and had not seen. However, they also falsely claimed to have seen 26% of the missing clips, or an additional 13.5 s of the event. Moreover, participants were more likely to falsely remember seeing the cruxes, the more traumatic scenes, compared to the non-cruxes.

Drawing on the SMF, we proposed that there are at least two, possibly related, routes to the pattern of memory distortion we observed, both of which rely on mental imagery (3, 4). First, we argued that it is possible participants recognized that there were gaps in the film and intentionally generated imagery – that echoed the content of the missing clips – to fill those gaps (4). Second, we argued that it is also possible participants did not notice the gaps in the film and instead their intrusive thoughts and images about the film happened to echo the content of the missing clips [e.g., (19)]. Of course, these two routes are not mutually exclusive: both rely on source monitoring failures, both may involve conscious and unconscious elements, and therefore both are likely to play a role in distorting participants’ memories for the film. Nevertheless, we argued that if source monitoring errors were responsible for the memory distortion we observed, we should be able to manipulate the likelihood of those errors by encouraging different approaches to source monitoring.

Therefore, in a subsequent study, using the same film, we first drew attention to the gaps at encoding and then attempted to induce different approaches to source monitoring (26). Specifically, some participants saw visual static – just like the “snow” on an untuned television – for the duration of the missing clips. This static clearly identified that the film was missing particular scenes. We compared the memory performance of those participants to the performance of participants who simply saw our original film, which did not highlight the missing scenes (2). Here, we found that highlighting the missing scenes had no impact on the pattern of memory distortion we observed.

However, we included two further conditions – where participants also saw visual static highlighting the missing scenes – to test the impact of different source monitoring strategies. First, we warned some participants before encoding that we had removed some scenes. The purpose of this warning was to encourage a more systematic source monitoring approach – a slower, more deliberate and controlled, style – when these participants came to evaluate what they had and had not seen at test. Indeed, basic memory research demonstrates the effectiveness of similar advanced warnings [e.g., (27, 28)]. Our results suggested the warning worked: warned participants exhibited less memory distortion than unwarned participants. Second, we also included a condition where participants saw a brief written description of the missing scenes overlaying the visual static. The purpose of this label was to specify the missing content so that participants could imagine what occurred between the depicted scenes. Related research has shown that the more detail people are given about a scenario, the easier it is for them to imagine that scenario (4, 29). Thus, we expected that if participants did generate mental imagery that fitted with the label, then the missing scenes would feel more familiar at test and participants would rely on a more heuristic – more rapid, non-deliberative, less controlled – source monitoring approach (4). Here too, the data supported our predictions: people exhibited more memory distortion when they saw a label specifying the missing content. Taken together then, our research provides indirect evidence that mental imagery plays a role in traumatic memory distortion.

Of course, there are significant methodological limitations to keep in mind when evaluating all laboratory-based research on traumatic memory. Although laboratory research can provide critical insights as a result of tightly controlled experimental designs, it is frequently a poor analog for an event that meets the criteria described in the Diagnostic and Statistical Manual of Mental Disorder’s (5th Ed.) Criterion A (30). For example, the stress and trauma-induction procedures researchers employ cannot ethically or morally reach the levels people experience in a real-world trauma. Moreover, participants are typically “witnesses” to an experience rather than the “victim” of the experience, the duration of the events is limited and delays are often truncated to meet experimental demands.

Nevertheless, we believe that developing a better understanding of source monitoring errors and the role of mental imagery in traumatic memory distortion should be a research priority. How people exposed to trauma remember and misremember aspects of their experiences in ways that influence their recovery is both theoretically and practically important. For example, the touted correlation between the likelihood a person will develop PTSD and the severity of their experienced trauma is largely based on observed correlations between self-reported current symptoms and retrospective reports about the severity of the trauma [e.g., (1, 19)]. That relationship – likely distorted and exacerbated by a person’s current memory for the event – could well be masking other, better predictors of PTSD. Thus, to determine the true psychological impact of trauma, and therefore the best ways to treat maladaptive reactions to that trauma, we must know to what extent memory (in)accuracy plays a role. Hence, we will continue to investigate the extent, causes, and triggering conditions of errors in memory for traumatic experiences.

## Conflict of Interest Statement

The authors declare that the research was conducted in the absence of any commercial or financial relationships that could be construed as a potential conflict of interest.
